# Activation Mechanisms of Natural Killer Cells during Influenza Virus Infection

**DOI:** 10.1371/journal.pone.0051858

**Published:** 2012-12-31

**Authors:** Ilwoong Hwang, Jeannine M. Scott, Tejaswi Kakarla, David M. Duriancik, Seohyun Choi, Chunghwan Cho, Taehyung Lee, Hyojin Park, Anthony R. French, Eleni Beli, Elizabeth Gardner, Sungjin Kim

**Affiliations:** 1 Department of Microbiology and Molecular Genetics, Michigan State University, East Lansing, Michigan, United States of America; 2 Department of Food Science and Human Nutrition, Michigan State University, East Lansing, Michigan, United States of America; 3 Division of Pediatric Rheumatology, Department of Pediatrics, Washington University School of Medicine, St. Louis, Missouri, United States of America; McMaster University, Canada

## Abstract

During early viral infection, activation of natural killer (NK) cells elicits the effector functions of target cell lysis and cytokine production. However, the cellular and molecular mechanisms leading to NK cell activation during viral infections are incompletely understood. In this study, using a model of acute viral infection, we investigated the mechanisms controlling cytotoxic activity and cytokine production in response to influenza (flu) virus. Analysis of cytokine receptor deficient mice demonstrated that type I interferons (IFNs), but not IL-12 or IL-18, were critical for the NK cell expression of both IFN-γ and granzyme B in response to flu infection. Further, adoptive transfer experiments revealed that NK cell activation was mediated by type I IFNs acting directly on NK cells. Analysis of signal transduction molecules showed that during flu infection, STAT1 activation in NK cells was completely dependent on direct type I IFN signaling, whereas STAT4 activation was only partially dependent. In addition, granzyme B induction in NK cells was mediated by signaling primarily through STAT1, but not STAT4, while IFN-γ production was mediated by signaling through STAT4, but not STAT1. Therefore, our findings demonstrate the importance of direct action of type I IFNs on NK cells to mount effective NK cell responses in the context of flu infection and delineate NK cell signaling pathways responsible for controlling cytotoxic activity and cytokine production.

## Introduction

NK cells are innate lymphocytes that have potent activity for controlling viral infections through the production of cytokines and the direct killing of infected target cells [1], [2], [3]. The importance of NK cell antiviral activity was first appreciated when an individual with high susceptibility to recurring herpesvirus infection was found to be deficient for NK cells [4]. Since this discovery, numerous studies have demonstrated strong association between NK cell activity and the control of herpesviruses, including human cytomegalovirus (CMV) in particular [1], [5], [6]. Studies aimed at a better understanding of the interaction between CMV and human immune cells have also revealed that the virus has developed elaborate means of evading detection by NK cells [3], [7], [8], [9], further demonstrating the importance of NK cell activity for protection of the host. Much of our understanding of how NK cells control CMV has come from studies using murine CMV (MCMV), and includes the identification of MCMV m157, a key ligand that binds to the NK cell activation receptor Ly49H [10], [11]. This receptor is required for resistance to MCMV in vivo, and through this receptor-ligand pair, NK cells are activated to produce IFN-γ and undergo proliferation [12], [13]. Though less well-studied, NK cells are also important for controlling infections by other herpesviruses including herpes simplex virus (HSV), Epstein-Barr virus, human herpesvirus-6, [14], [15], [16], [17] and non-herpesviruses, including vaccinia virus, hepatitis B and C viruses, and HIV [18], [19], [20], [21], [22]. However, much less is known about how NK cells respond to other viruses, including influenza virus, an important human pathogen that causes substantial morbidity and mortality worldwide [23].

Influenza viruses are enveloped, negative-strand RNA viruses that are transmitted through contact with infected individuals or contaminated items, and through inhalation of aerosols, leading to seasonal outbreaks of acute respiratory tract infection, for which yearly vaccination, if available, can provide some measure of protection. However, rapid alterations in antigenic properties can yield emerging variants of influenza with the potential to cause pandemic infections [23], [24]. Whether NK cells play an important role in the control of influenza virus infection has been controversial. Accumulating evidence supporting the importance of NK cell activity against influenza includes studies in which mice with reduced numbers of NK cells, as with antibody-induced NK cell depletion, have higher morbidity and mortality associated with flu infection [25]. Alternatively, mice with hyper-responsive NK cells are more resistant to flu [26]. Also, susceptibility to flu infection is higher in mice with certain genetic deficiencies, including deficiency of the cytolytic molecule perforin, or the natural cytotoxicity receptor NKp46 that recognizes influenza hemagglutinins on infected target cells [27], [28]. Additionally, we have shown that aged mice with reduced lung and splenic NK cells are more susceptible to flu infection, primarily due to reduced NK cell functionality in response to activation stimuli [29].

With respect to human infection, additional evidence pointing toward the importance of NK cell antiviral activity against flu infection is described in a report of clinical cases of severe infection by new flu subtypes that coincided with significantly reduced NK cells [30]. In the most severe case, which ended in lethality, viral nucleic acid was detected in the patient plasma, illustrating the ability of flu to establish systemic as well as respiratory infections [31], [32]. In contrast, a recent study reported that mice had significantly less lung lesions and pro-inflammatory cytokines in bronchiolar lavage fluid during flu infection when they were either depleted of NK cells, or deficient for IL-15, a cytokine important for the maintenance of NK cells [33]. This study suggested that in some settings, NK cells might contribute to the pathogenesis of influenza in the lungs. However, these studies used different strains of mice, which may have different susceptibilities to the particular strain of flu that was used. Nonetheless, in all cases, whether contributing to the control or the pathogenesis of infection, NK cells were activated in response to flu infection. However, mechanisms controlling NK cell activation during flu infection remain poorly understood.

Among the cytokines produced during the early stages of viral infection are type I IFNs, a family of cytokines consisting of multiple members that all act through the type I IFN receptor (IFNAR), a heterodimeric receptor expressed on a wide variety of cells. Type I IFNs are known to play important roles in antiviral defense by regulating immune function, including the activation of NK cells for cytotoxic activity and IFN-γ production in both human and mouse NK cells [34], [35], [36], [37], [38], [39], [40], [41]. Depending on the pathogen and TLR agonists present, the activation of the NK cells by type I IFNs can be either direct, e.g. via interaction with type I IFN receptor expressed on NK cells, or indirect, e.g. via production of activating cytokines by other type I IFN-responsive immune cells, such as the production of IL-15 by dendritic cells [41], [42], [43], [44]. During vaccinia virus infection, NK cells are activated either directly by type I IFNs [43] or indirectly through binding of various TLR agonists [42]. Furthermore, it has been reported recently that NK cell activation following stimulation with polyinosinic-polycytidylic acid, a TLR3 agonist, involves both direct and indirect action of type I IFNs [44]. In the context of influenza infection, recognition of the virus by innate immune cells activates the TLR7–MyD88 pathway, and leads to the rapid and abundant production of type I IFNs (IFN-α/β) by plasmacytoid dendritic cells [45], [46], [47]. Currently, the role of type I IFNs for specific aspects of NK cell effector functions during influenza infection remains unclear. Furthermore, during flu infection, the contribution of other cytokines to NK cell activation is also poorly understood.

In addition to type I IFNs, several other cytokines elicited during early viral infection contribute to the control of the infection. Studies have shown that, along with type I IFNs, IL-12, IL-15 and IL-18 are major components of NK cell activation during viral infections [1], [2], [3], [48]. For instance, IL-12, and IL-18 are required for IFN-γ production by NK cells during MCMV infection although compartmental differences for IL-18 have been noted [38], [49], [50], [51]. Likewise, IL-18 is required during HSV-1 infection for the IFN-γ response of NK cells [52]. Another important mechanism of antiviral activity is up-regulation of the cytolytic effector molecules, which can be induced in NK cells following MCMV and vaccinia virus infection, mediated by IL-15 and type I IFNs, respectively [43], [53]. While much of our current understanding of the pathways leading to NK cell activation during viral infection has been gained primarily through studies involving MCMV infection, different viruses may use different pathways for NK cell activation, and the importance of NK cells, as well as the cytokine signaling pathways and mechanisms for NK cell activation during infection by influenza virus are not well understood. Furthermore, whether NK cell responsiveness to influenza requires activation of specific cytokine pathways is not clear.

Type I IFNs and IL-12 activate NK cell effector responses via signaling through the JAK-STAT pathway. However, the axes of activation can differ depending on the pathogen. For example, during MCMV infection, IL-12 mediates activation of STAT4 for IFN-γ production by NK cells [38], [49], [50]. On the other hand, during lymphocytic choriomeningitis virus (LCMV) infection, STAT4 activation for the IFN-γ response of NK cells is mediated by type I IFN signaling [41], [54], [55]. In addition, target cell killing by NK cells during viral infection was poor in the absence of STAT1, suggesting that Type I IFNs and STAT1 are important for NK cytolytic activity; however, the mechanism of this relationship remains unclear. It has been reported that NK cells produce IFN-γ during flu infection as well [26]. However, the signaling pathways involved in this infection response are not well understood.

While MCMV and LCMV have served as model pathogens to study NK cell activation during viral infection, studies regarding the responses of NK cells during influenza infection have been hampered due to low numbers of responding cells in murine lungs. To facilitate our investigation into the mechansims controlling NK cell activation during influenza infection, we developed a systemic model of acute influenza infection which allowed the analysis of NK cell functional responses and signaling during early infection. In this study, we have also used adoptive transfer experiments to further delineate the cellular and molecular pathways of NK cell activation during influenza infection.

## Materials and Methods

### Mice

C57BL/6 mice (B6) and CD45.1^+^ B6 (carrying the Ly5.1 allele) mice were purchased from the National Cancer Institute. IL-12R^−/−^ or IL-18R^−/−^ (B6 background), STAT4^−/−^ (BALB/c background), and BALB/c WT mice were purchased from The Jackson Laboratory. STAT1^−/−^ (129/Sv background) and 129/Sv were purchased from Taconic Farms. IFNAR^−/−^ mice were on the B6 background [56]. In some experiments, IFNAR^+/−^ littermates were used as controls. Mice were housed in specific pathogen free facilities, and all animal experimental protocols were approved by the Institutional Animal Care and Use Committee at Michigan State University.

### Antibodies and Flow Cytometry

Antibodies against the following proteins were purchased as indicated: NK1.1 (PK136), CD3 (145-2C11), CD19 (1D3), IFN-γ (XMG1.2), granzyme B (GB11), CD107a (1D4B), CD69 (H1.2F3), pSTAT1 pY701 (4a) and pSTAT4 pY693 (38/p-Stat4) from BD Biosciences, CD49b (DX5) from eBiosciences, and CD45.2 (104) from Biolegend. Anti-CD16/32 was included for all surface marker staining to block non-specific antibody binding and was present in culture supernatant of the hybridoma cell line 2.4G2 [57]. Flow cytometry was performed using an LSR II instrument (BD) and data were analyzed with FlowJo software (Tree Star, Inc.).

### Virus Infection and Adoptive Transfer

**Figure 1:**
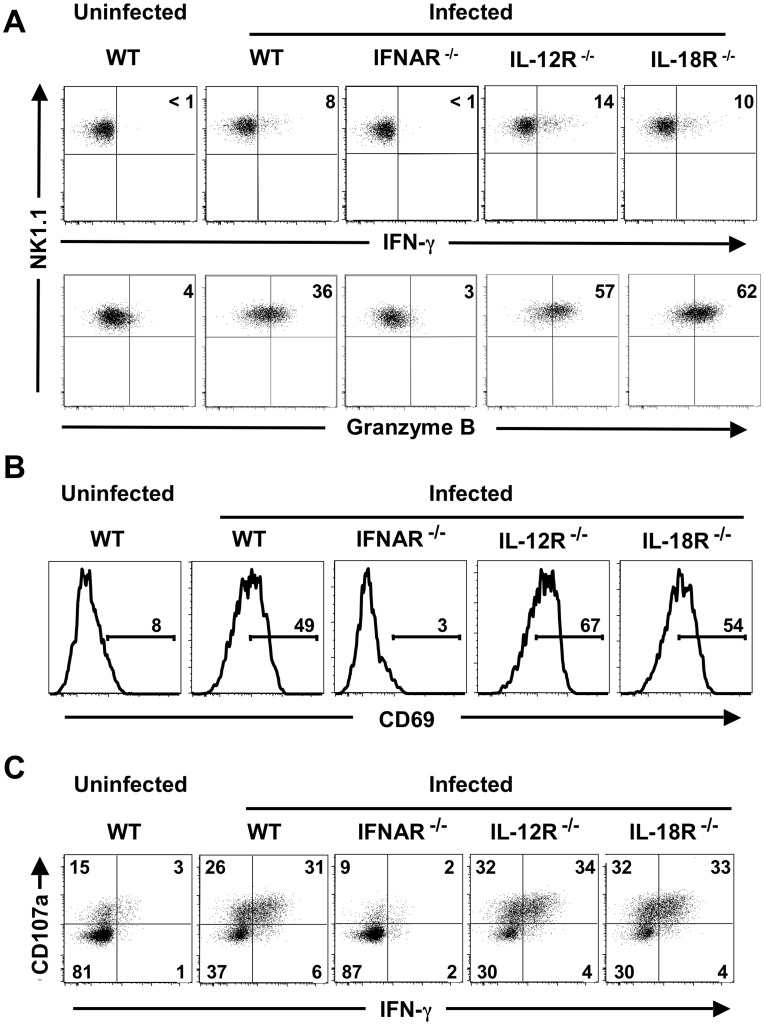
Type I IFNs are required for NK cell activation in response to flu infection. Following i.v. infection with flu, splenic NK cells (NK1.1**^+^**CD3**^−^**CD19**^−^**) from B6 WT, IFNAR**^−/−^**, IL-12R**^−/−^** and IL-18R**^−/−^** mice were analyzed at 9h post-infection. (A) IFN-γ and granzyme B expression are shown. Mouse genotypes are indicated above the dot plots or histograms. Inset values represent the percentages of IFN-γ**^+^** (upper panels) or granzyme B**^+^** (lower panels) NK cells. (B) CD69 expression levels on NK cells from uninfected and infected mice are shown. Percentages of NK cells located within the CD69**^+^**gate are indicated. (C) CD107a expression and IFN-γ production were analyzed in NK cells after incubation with YAC-1 cells. Percentages of NK cells within each quadrant are indicated. Data are representative of at least three mice per group.

**Figure 2:**
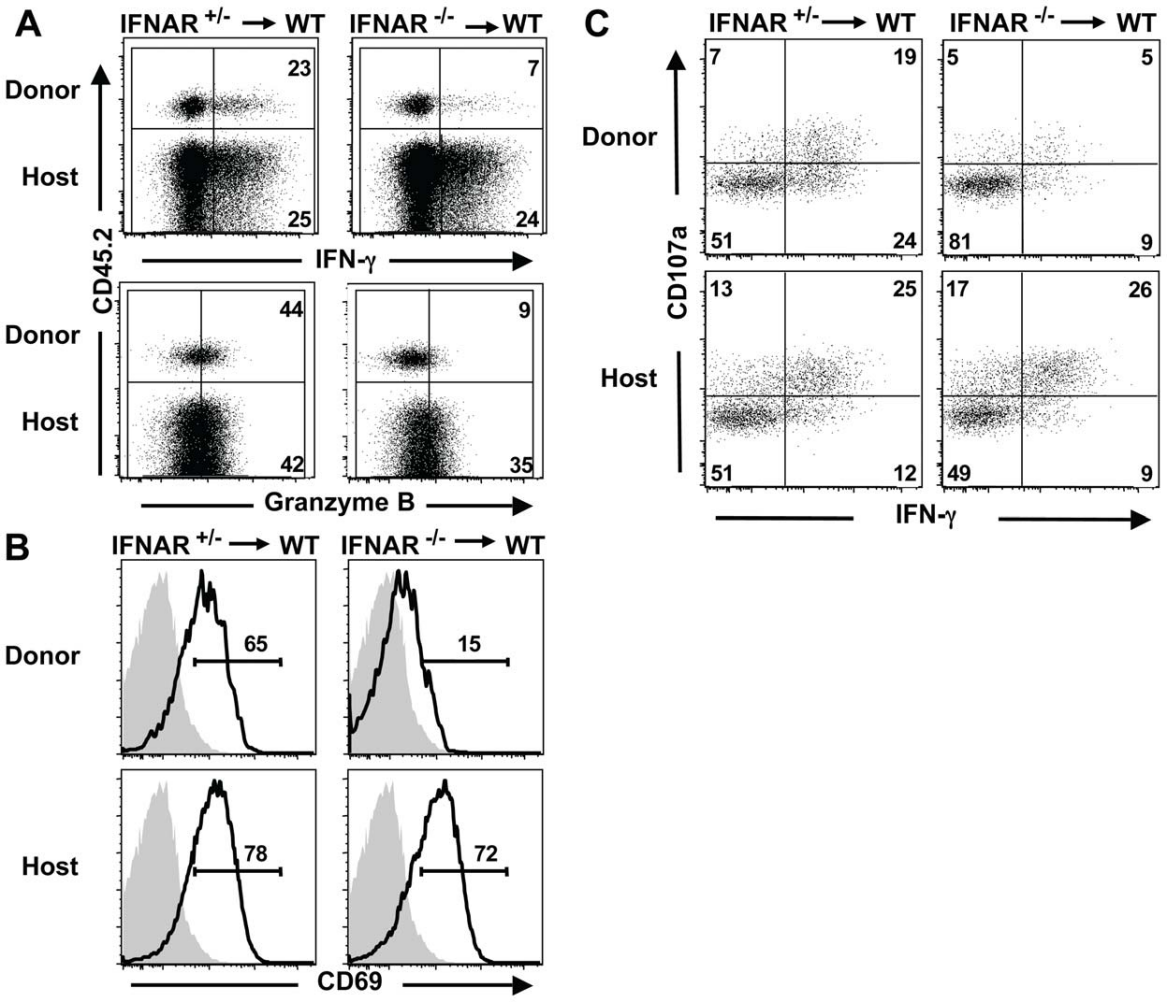
Direct action of type I IFNs is critical for activation of NK cells following flu infection. Splenocytes from IFNAR**^+/−^** or IFNAR**^−/−^** (CD45.2**^+^**) were transferred into CD45.1**^+^** B6 WT recipients by i.v. injection prior to infection with flu. Splenic NK cells from indicated mice were analyzed post-infection. (A) Expression levels of IFN-γ (upper panels) and granzyme B (lower panels) were analyzed in NK cells after transfer and infection. Donor and recipient genotypes are indicated, and upper and lower quadrants for each dot plot represent the donor and host NK cells, respectively. Inset values indicate the percentages of IFN-γ**^+^** or granzyme B**^+^** NK cells. (B) CD69 expression of NK cells from infected (open histograms) and uninfected (shaded histograms) mice. Percentages of NK cells located within the CD69**^+^**gate are indicated. (C) IFN-γ and CD107a expression were analyzed after in vitro stimulation of splenocytes with YAC-1 cells. Numbers represent the relative percentages of CD107a**^+^** and IFN-γ**^+^** NK cells for donor and host cells. Data are representative of four separate experiments with 2–4 mice per group.

Experiments involving influenza infection used the mouse-adapted strain of H1N1 influenza A/Puerto Rico/8/34 (A/PR8) [29]. For in vivo experiments, mice were infected by intravenous (i.v.) injection of 100 hemagglutinin units (HAU) via the retro-orbital sinus for all experiments except Figures 1 and 2A (IFN-γ), for which 500 HAU was used. Animals were sacrificed 9 h post-infection (hpi) for NK cell analysis. For pulmonary NK cell testing, mice were anesthetized with avertin (2,2,2-tribromoethanol, Sigma-Aldrich) then infected intranasally (i.n.) with 16 HAU and sacrificed 2 d after infection. Doses for each route of infection were determined based on dose-response curves to optimize responsiveness for each preparation of virus. In vitro infections were performed by culturing splenocytes (20–60×10^6^/ml) with 100 HAU/mL virus for 6 h to evaluate signaling or 8 h plus an additional 4 h in the presence of brefeldin A for effector molecule and functional assays. In some experiments, CD45.1^+^ and CD45.2^+^ splenocytes were combined in equal numbers just prior to in vitro infection with virus. For adoptive transfer, splenocytes (30–50×10^6^) from CD45.2^+^ IFNAR^+/−^ or CD45.2^+^ IFNAR^−/−^ mice were injected i.v. into CD45.1^+^ B6 recipient mice 2 days prior to virus infection.

### Cytokine and Degranulation Assays

Splenocytes and lung NK cells were prepared as previously described [29], [58]. IFN-γ production was detected by incubating splenocytes in vitro for 4 h in the presence of brefeldin A. For granzyme B analysis, splenic NK cells were directly stained with FACS antibodies without additional incubation. For degranulation assays, splenocytes were mixed with YAC-1 tumor target cells (E:T  = 10∶1) in the presence of anti-CD107a and monensin for 4 h. In preparation for flow cytometric analysis, cells were first stained to identify surface markers, then fixed and permeabilized, followed by intracellular staining of effector molecules. NK1.1 or CD49b were used to identify NK cells. Cells that expressed CD3 and CD19 were excluded from NK cell analysis in all experiments.

### Signaling Molecule Assays

Experiments were performed as previously described [59]. Splenocytes were harvested from either infected or naïve mice, or cultured in vitro with virus, as indicated, prior to fixation with formaldehyde and permeablization with ice-cold methanol. Cell surface markers and intracellular phospho-STAT (pSTAT) proteins were stained and analyzed using flow cytometry.

### ^51^Cr Release Assays

Splenic NK cells were enriched by negative selection (Miltenyi Biotec), then NK1.1^+^CD3**^−^**CD19**^−^** cells were sorted using a FACS Vantage cytometer (BD). NK cells were incubated in the presence of IL-15 (50 ng/mL, PeproTech), IL-18 (100 ng/mL, MBL), or IFN-α (1,000U/mL, PBL InterferonSource), or in the absence of cytokines for 6 h and washed with warm culture medium three times, then co-cultured with ^51^Cr-labeled YAC-1 cells for 4 h in triplicate wells at indicated ratios. Specific lysis was calculated as described previously [60]. Mean ± SEM values were determined using GraphPad Prism software.

### Statistics

Statistical analyses were performed using the unpaired Student’s t-test. Differences were considered statistically significant when *P*<0.05.

## Results

### Requirement of Type I IFNs for NK Cell Activation during Influenza Infection

To explore mechanisms for NK cell activation in response to influenza, we first tested the possibility of whether flu infection in vitro leads to detectable NK cell cytokine production and increased cytolytic potential. When splenocytes from wild type (WT) mice were cultured in the presence of influenza virus, the NK cells produced significant amounts of IFN-γ, which peaked between 8 and 12 hours (Figure S1 and data not shown), indicating NK cell antiviral activity can be induced in vitro. Similarly, substantial granzyme B expression was also detected after 8 hours. Thus, these data demonstrate that murine NK cells can vigorously respond to influenza virus in vitro.

Next, we investigated the roles of cytokines for IFN-γ production and granzyme B induction by NK cells to influenza infection in vivo. Several cytokines, including type I IFNs, IL-12, IL-15 and IL-18 are known to be important for NK cell activity against viral pathogens [1], [2], [3], [48], but their roles during influenza infection specifically are not clear. For this purpose, we used WT mice or mice that were deficient for either type I IFN, IL-12, or IL-18 receptors. IL-15 receptor-deficient mice couldn’t be tested due to the absence of NK cells in these animals [61]. Following acute i.v. flu infection, we found that both IFN-γ and granzyme B expression levels in NK cells from infected WT mice were increased compared to uninfected animals similar to the in vitro infection data at 8–12 hpi (Figures 1A and S2). NK cell responsiveness was also evaluated after longer periods of infection (1, 2 and 3 days post-infection), but IFN-γ production was much less or nearly undetectable (data not shown). Thus, shorter time points, similar to the in vitro experiments, were used for the in vivo studies. We then compared the NK cell responsiveness of mice that lack receptors for type I IFNs, IL-12 or IL-18 to that of B6 WT mice. NK cells from infected IFNAR^−/−^ mice showed no detectable increase in IFN-γ or granzyme B over the levels observed for the uninfected control mice (Figures 1A and S2). In contrast, NK cells from infected IL-12R^−/−^ and IL-18R^−/−^ mice displayed similar, or even higher levels of these effector molecules compared to NK cells from infected WT mice. In addition, we measured the expression of the activation marker CD69 and found NK cells from flu-infected WT, IL-12R^−/−^ and IL-18R^−/−^ mice displayed increased levels of CD69, whereas CD69 levels on NK cells from infected IFNAR^−/−^ mice were similar to those observed for uninfected controls (Figure 1B).

To further assess the requirement of these cytokines for NK cell responsiveness to target cells in the context of flu infection, we evaluated the NK cell cytotoxic potential and IFN-γ production following incubation with YAC-1 tumor cells. NK cells obtained from infected WT mice showed markedly higher levels of IFN-γ and CD107a, a marker of cytotoxic degranulation, compared to those from uninfected controls (Figure 1C). However, NK cells from infected IFNAR^−/−^ mice showed no increase in the levels of either IFN-γ or CD107a compared to uninfected controls, while NK cells from infected IL-12R^−/−^ and IL-18R^−/−^ mice exhibited fully-enhanced responsiveness following incubation with YAC-1 cells.

Since the most common route of infection is via the respiratory tract, we also wanted to confirm the NK cell data after intranasal infection, and found that, although the effector molecule expression levels were quite low, the patterns were consistent with the splenic NK cell results, i.e. pulmonary NK cells from IFNAR^−/−^ mice did not produce IFN-γ or granzyme B after i.n. inoculation with flu (Figure S3). A limitation of this infection route is that IFN-γ production by lung NK cells at 2 days was detectable in only 2–3% of the recoverable NK cells, consistent with a previous study [29], while only ∼10% of the lung NK cells show granzyme B expression (Figure S3). In addition to the low level of induction and low numbers of recoverable NK cells from lung tissue, this infection route presents technical challenges for studying potentially subtle changes in biochemical signaling pathways. Thus, the i.v. infection route provided a method for simultaneous exposure of a substantial percentage of splenic NK cells, and facilitated the analysis of NK cell functional activity and factors pertaining to signaling pathways that are involved in NK cell responses to flu infection. Taken together, these data suggest that type I IFNs are required for both NK cell IFN-γ and granzyme B production during flu infection, whereas the contributions of IL-12 or IL-18 are negligible.

### Direct Action of Type I IFNs is Required for NK Cell Activation during Flu Infection

NK cell priming and/or activation mediated by type I IFNs in response to TLR agonists or pathogens other than flu have been shown to occur through either direct action on the cells (e.g. via the type I IFN receptor) [41], [43] or indirect action, such as through type I IFN-mediated trans-presentation of IL-15 by dendritic cells [42], or both [44]. To determine whether the type I IFN-mediated effects on NK cell functional activity during flu infection is due to direct or indirect action, we used adoptive transfer of IFNAR^−/−^ or WT splenocytes (both carrying the CD45.2 allele) into B6 WT (CD45.1^+^) host mice, followed by i.v injection of flu. Upon sacrifice, donor and host-derived splenic NK cells were identified according to the expression of this allelic marker. Analysis of intracellular effector molecules showed that WT donor NK cells produced IFN-γ and granzyme B at levels equivalent to the WT host NK cells (Figure 2A, left panels and Figure S4). In contrast, despite the WT environment containing type I IFNs and other cytokines such as IL-15, NK cells derived from the IFNAR^−/−^ responded poorly, as the expression levels of these effector molecules were much lower than those from the WT donor NK cells (Figure 2A). Similarly, up-regulation of CD69 expression on NK cells derived from IFNAR^−/−^ donor mice was impaired during flu infection (Figure 2B). Therefore, NK cells that lack the receptor for type I IFNs exhibit severely impaired activation in WT environments, demonstrating that direct action of type I IFNs plays a dominant role in NK cell responsiveness during acute flu infection.

We tested the capacity of the transferred NK cells to respond to further stimulation after adoptive transfer and flu infection by analyzing donor and host NK cell IFN-γ production and degranulation activity following incubation with YAC-1 cells. NK cells derived from the WT donor expressed IFN-γ and CD107a after YAC-1 stimulation at levels comparable to the NK cells derived from the WT host (Figure 2C, left panels). In contrast, the transferred IFNAR^−/−^ NK cells exhibited much lower expression of these effector molecules in response to YAC-1 cells compared to the WT donor or host NK cells (Figure 2C). Thus, direct action of type I IFNs during influenza infection is required to achieve full functional activity for cytokine production and degranulation in response to target cells or cytokine stimulation.

**Figure 3:**
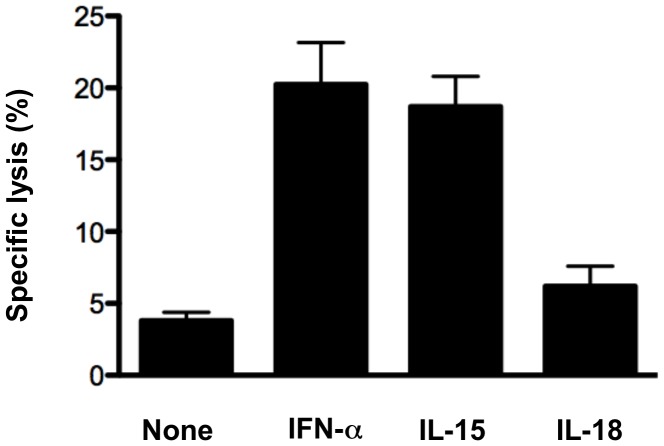
Highly purified NK cells exhibit enhanced cytotoxicity toward target cells in response to treatment with IFN-α. Sorted NK cells (>99% pure) were cultured in vitro for 6 hours with or without indicated cytokines, then combined with ^51^Cr-labeled YAC-1 targets (E:T = 10∶1) to assess cytotoxic activity. Bars represent the average specific lysis of target cells for each condition. Error bars denote the SEM. Data are representative of two independent experiments.

To address whether cytolytic activity of NK cells was enhanced by type I IFNs alone, or if the enhancement was dependent on contributing factors from other cell types, splenocyte preparations were sorted to obtain highly purified populations of NK cells from uninfected B6 WT mice. The sorted cells were pre-treated with IFN-α, IL-15 and IL-18 prior to incubation with ^51^Cr-labeled YAC-1 target cells. Analysis of specific lysis indicated that IFN-α-treated NK cells exhibited target cell killing at levels equivalent to IL-15-treated NK cells, whereas IL-18 had little effect (Figure 3). While it is possible that a longer incubation could lead to further increases in killing activity, we found this was not practical as sorted NK cells have poor survival when cultured in the absence of IL-15 or IL-2 for extended periods of time. Thus, our findings indicate that direct action of IFN-α on NK cells is sufficient to enhance NK cell killing activity. Collectively, our data demonstrate that direct action of type I IFNs is critical for NK cell responsiveness to influenza infection.

### NK Cells are Dependent on Type I IFNs for STAT1 Activation during Influenza Infection

**Figure 4:**
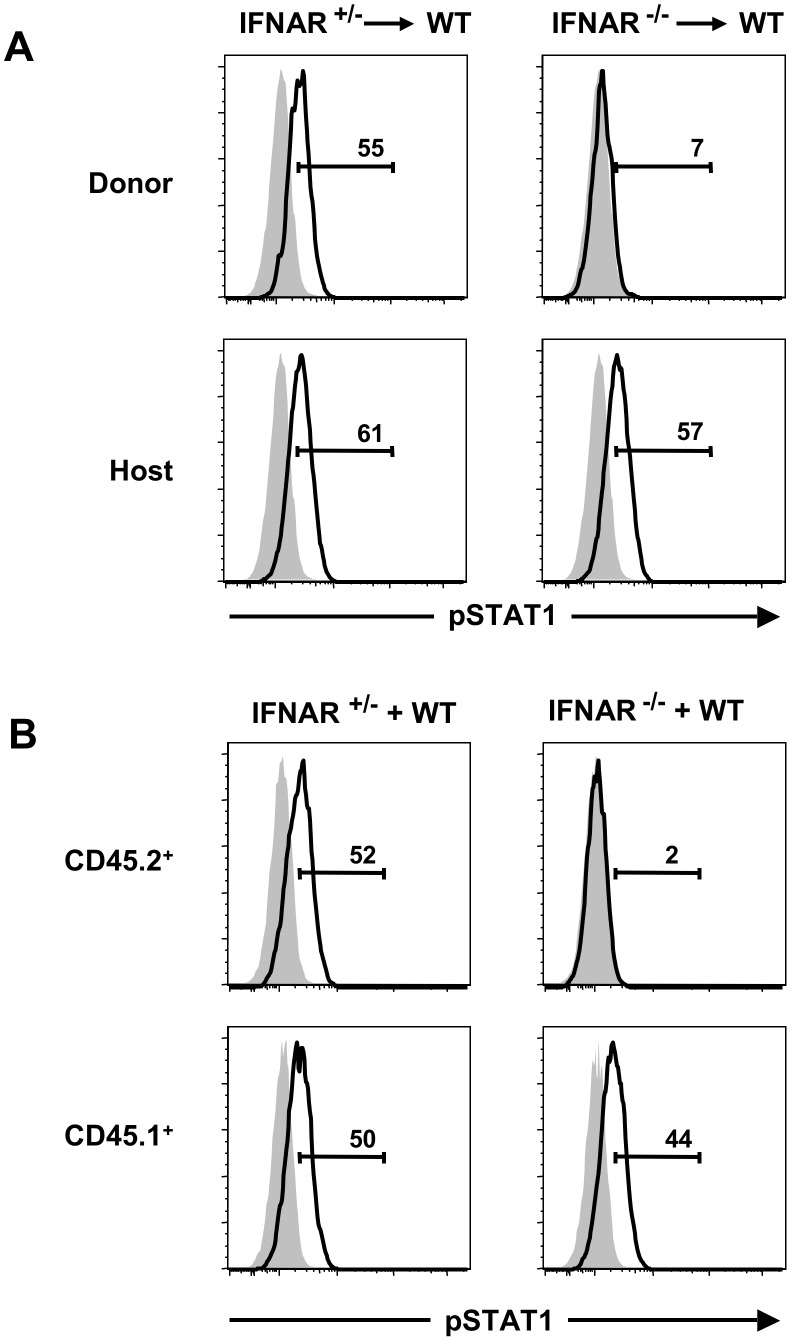
Activation of STAT1 in NK cells requires the direct action of type I IFNs during influenza infection. NK cells from infected (open histograms) and uninfected (shaded histograms) mice were analyzed for intracellular pSTAT1 following adoptive transfer (A) or co-culture (B). Adoptive transfer was as described in Figure 2. For in vitro infection, CD45.2**^+^** splenocytes from IFNAR**^+/−^** or IFNAR**^−/−^** mice were combined with CD45.1**^+^** B6 splenocytes at 1∶1 ratio, then infected with flu. NK cells (NK1.1**^+^**CD3**^−^**) from infected (open histograms) and uninfected (shaded histograms) samples were analyzed for intracellular pSTAT1. Values represent the percentages of pSTAT1**^+^** NK cells. Data are representative of three independent experiments with 2–4 (A) or 1–3 (B) mice per group.

Type I IFNs activate NK cells through the JAK-STAT pathway, leading to phosphorylation of signalling proteins STAT1 and STAT4, which are important mediators of NK cell effector functions [41], [54], [55], [62]. We therefore sought to determine whether activation of STAT proteins in NK cells during flu infection was through the direct or indirect action of type I IFNs. For this experiment, we transferred CD45.2^+^ splenocytes from IFNAR^+/−^ or IFNAR^−/−^ donor mice into B6 WT hosts (CD45.1^+^), followed by infection with influenza, and phosphorylation analysis of STAT1. NK cells derived from the IFNAR^+/−^ donors displayed levels of pSTAT1 similar to WT host-derived NK cells following flu infection (Figure 4A, left panels). In contrast, activation of STAT1 in IFNAR^−/−^ donor-derived NK cells after flu infection, or IFN-α, was severely abrogated (Figures 4A and S5). Similar results were obtained in vitro by combining splenocytes from IFNAR^+/−^ or IFNAR^−/−^ (CD45.2^+^) mice with splenocytes from B6 WT (CD45.1^+^) mice, followed by infection with flu (Figure 4B). Therefore, in both in vivo and in vitro analyses, the activation of STAT1 in IFNAR^−/−^ CD45.2^+^ NK cells was completely abrogated, indicating that the direct action of type I IFNs has a significant impact on STAT1 activation in NK cells following flu infection.

**Figure 5:**
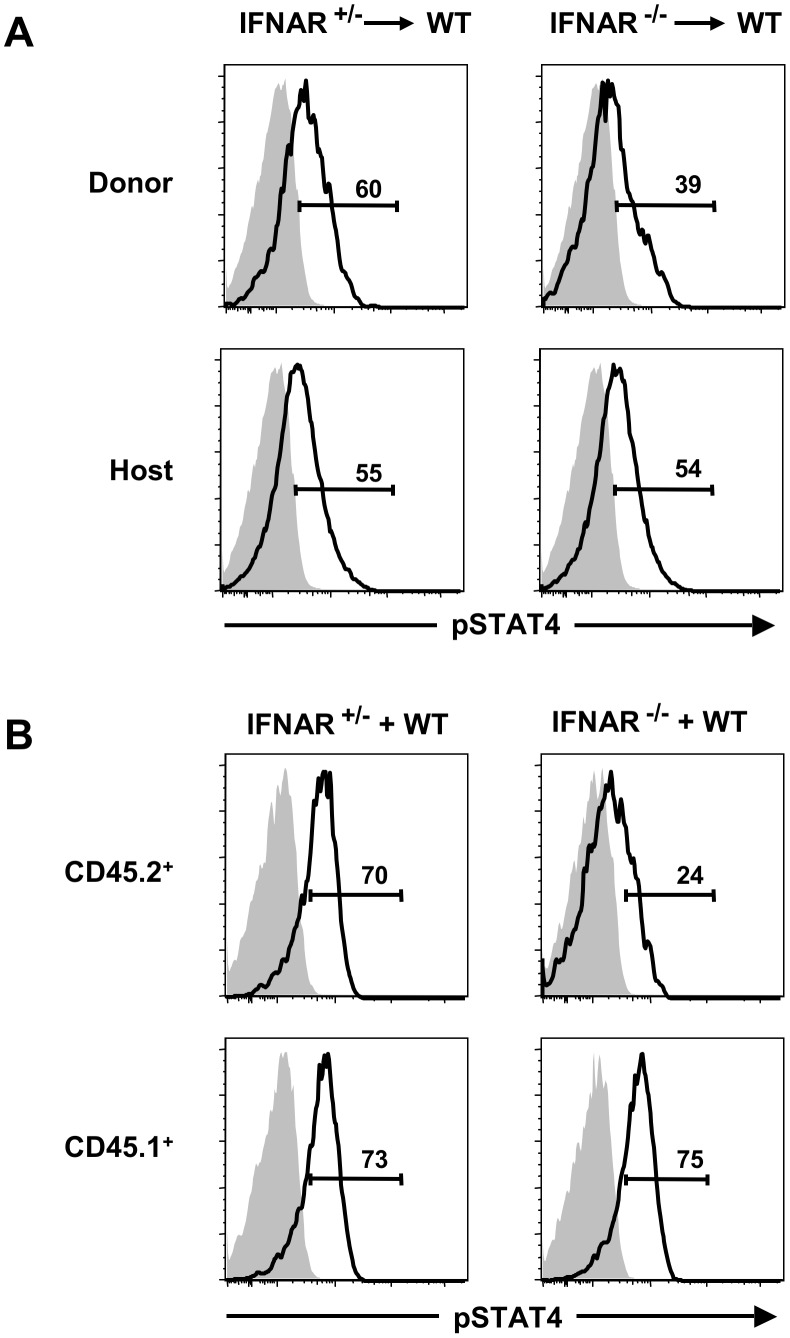
Activation of STAT4 is partially dependent on the direct action of type I IFNs during influenza infection. NK cells from infected (open histograms) and uninfected (shaded histograms) mice were analyzed for intracellular pSTAT4 following adoptive transfer (A) or co-culture (B). Adoptive transfer was as described in Figure 2. For in vitro infection, CD45.2**^+^** splenocytes from IFNAR**^+/−^** or IFNAR**^−/−^** mice were combined with CD45.1**^+^** B6 splenocytes at 1∶1 ratio, then infected with flu. NK cells (NK1.1**^+^**CD3**^−^**) from infected (open histograms) and uninfected (shaded histograms) samples were analyzed for intracellular pSTAT4. Values represent the percentages of pSTAT4**^+^** NK cells. Data are representative of three independent experiments with 2–4 (A) or 1–3 (B) mice per group.

Experiments to evaluate STAT4 activation following flu infection were also performed. Unlike the impaired activation of STAT1 in IFNAR^−/−^ NK cells, STAT4 was activated to an intermediate level in the absence of direct action of type I IFNs (Figure 5A). Combining splenocytes from IFNAR^+/−^ or IFNAR^−/−^ (CD45.2^+^) mice with splenocytes from B6 WT (CD45.1^+^) mice in vitro, followed by infection with flu yielded similar results, i.e. STAT4 was activated to an intermediate level in IFNAR^−/−^ NK cells (Figure 5B). These results suggest that during flu infection, cytokines other than type I IFNs, such as IL-12, may induce STAT4 activation in NK cells. Thus, direct action of type I IFNs has at least a partial impact on STAT4 activation since in the absence of type I IFN signaling, STAT4 activation in NK cells did not reach the levels observed in the WT host cells.

### STAT1 and STAT4 Mediate Differential NK Cell Effector Molecule Expression during Influenza Infection

**Figure 6:**
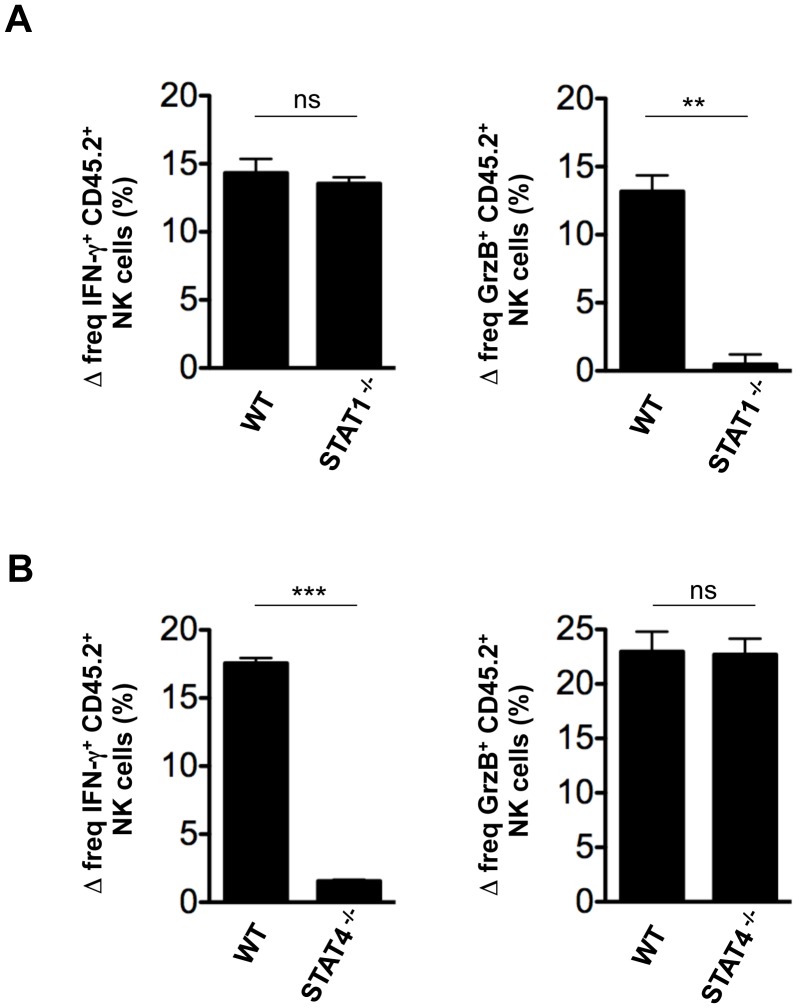
STAT1, but not STAT4, is required for granzyme B induction by NK cells in response to flu infection. CD45.2**^+^** splenocytes from 129/Sv WT and STAT1^−/−^ (A) or from BALB/c WT and STAT4^−/−^ (B) mice were combined with CD45.1**^+^** B6 splenocytes in vitro, then infected with flu. NK cells (DX5**^+^**CD3**^−^**CD19**^−^**) were analyzed for expression levels of IFN-γ (left panels) and granzyme B (right panels). Bar graphs represent the mean differences in percentage of IFN-γ**^+^** or granzyme B**^+^** CD45.2**^+^** NK cells from infected samples over uninfected controls. Error bars represent the SEM of triplicate samples. Data are representative of four independent experiments. **, *P*<0.001; ***, *P*<0.0001; ns, not significant.

We next investigated the potential roles of STAT1 and STAT4 in NK cell effector molecule expression during flu infection. Because STAT1 and STAT4 genetic knockout mice were not of the B6 background, adoptive transfer experiments could induce severe immune reactions, including NK cell activation. Therefore, we performed experiments in vitro by combining splenocytes (CD45.2^+^) from 129/Sv WT or STAT1^−/−^ mice with those from B6 (CD45.1^+^) mice, followed by influenza infection. Since each strain could yield different viral or cytokine responses, we used WT samples that were genetically matched to the STAT^−/−^ samples, in combination with B6 WT splenocytes, which served as the internal control. Upon analysis of CD45.2^+^ NK cells, we found that IFN-γ production by STAT1^−/−^ NK cells was not affected in the absence of STAT1 signaling. In contrast, granzyme B induction under these conditions was severely impaired (Figure 6A), indicating that STAT1 is required for granzyme B induction following flu infection. In a similar manner, we combined splenocytes (CD45.2^+^) from BALB/c WT or STAT4^−/−^ mice with B6 splenocytes (CD45.1^+^) in vitro, followed by flu infection. In contrast to STAT1^−/−^ NK cells, STAT4^−/−^ NK cells were defective for IFN-γ production, but displayed WT levels of granzyme B induction following influenza infection (Figure 6B). These results demonstrate that NK cells require STAT4 signaling for IFN-γ production, but not for granzyme B induction, during flu infection. Taken together, our data suggest that type I IFNs induce NK cell cytotoxic activity through the STAT1-granzyme B axis, but lead to IFN-γ production primarily through the STAT4 signaling pathway during flu infection.

## Discussion

In this study, we used an acute systemic infection model to investigate the role of type I IFNs in NK cell activation during flu infection. The i.v. route of infection was primarily chosen to circumvent the low number of activated NK cells found using the pulmonary infection route, as substantially larger percentages of NK cells could be impacted by the virus this way. Using this method, we found that type I IFNs play an important role in triggering both NK cell cytokine and cytolytic responses to influenza infection. Interestingly, however, NK cell activation was not compromised in the absence of IL-12 or IL-18 receptor signaling, both of which have been shown to activate NK cells during virus infection [48], [51], [52], [63], [64], [65], [66], [67].

Activation of NK cells by type I IFNs occurs through both direct and indirect mechanisms [41], [42], [43], [44]. Martinez et al. reported that NK cell activation following vaccinia virus infection was through the direct action of type I IFNs [43]. In contrast, NK cell activation can also occur through indirect action of type I IFNs on NK cells, such as through the trans-presentation of IL-15 by dendritic cells [42]. Pathogens may use different pathways to elicit cytokine production from accessory cells, and whether type I IFNs act directly or indirectly on NK cells in the context of influenza infection has been unknown. In this study, the IFNAR^−/−^ model allowed for investigation into this question since IFNAR^−/−^ NK cells could be adoptively transferred into a WT environment where accessory signals, such as cytokines produced by other immune cells, would be present in response to type I IFN secretion. From these adoptive transfer experiments, our data clearly point to a requirement for direct recognition of type IFNs by NK cells during flu infection, as the receptor-deficient NK cells exhibited severely impaired production of IFN-γ and granzyme B, as well as impaired cytotoxic potential, despite the WT environment. Therefore, the direct action of type I IFNs is likely to be a dominant mechanism for NK cell activation in response to flu infection. We cannot, however, completely rule out contributions from indirect pathways, such as IL-15 trans-presentation [42], [68], [69], [70], since we observed that transferred NK cells were slightly activated even in the absence of type I IFN signaling. Nonetheless, our data suggest that direct action of type I IFNs is a major factor in NK cell activation during early flu infection.

Direct stimulation of purified NK cells with IFN-α led to approximately four-fold greater cytotolytic activity compared to untreated controls. This is in contrast to a similar experiment in a previous report which showed NK cells were not primed directly by type I IFNs, and therefore, were unable to lyse target cells [42]. However, we noted that culturing of purified NK cells overnight, which was used in the previous report, led to significant loss of viability, presumably due to the requirement of IL-15 for NK cell survival [71], [72]. In contrast, when the culture time was limited to a shorter period (6 h), NK cell killing activity was equivalent to that observed following IL-15 priming. Therefore, our results indicate that even in the absence of other cell types, NK cells can respond well to the direct action of type I IFNs.

Our study has also shown that STAT1 and STAT4 in NK cells are activated during flu infection in a type I IFN-dependent manner. However, the dependence on type I IFNs was not equivalent for both STAT proteins, given that STAT1 activation of NK cells is fully dependent on type I IFNs, and STAT4 activation is only partially dependent, suggesting that other cytokines (e.g., IL-12) or accessory pathways might be involved in STAT4 activation by NK cells following flu infection [55], [73], [74], [75]. We also noted that full activation of STAT4 was not achieved in the absence of type I IFN signaling, highlighting the importance of direct action of type I IFN signaling for NK cell activation in vivo. STAT1 in NK cells can also be activated by cytokines other than type I IFNs, including IFN-γ [55], [75], [76], but our adoptive transfer experiments indicated that STAT1 is phosphorylated mainly through type I IFN signaling during flu infection. Thus, flu infection leads to type I IFN-mediated STAT1 activation and, possibly along with additional factors, STAT4 activation in NK cells.

Our data indicate that during flu infection STAT1 and STAT4 have differential roles in up-regulation of effector molecules in NK cells. We have shown that STAT1 is critical for the up-regulation of granzyme B, but not IFN-γ, in NK cells, suggesting that the axis comprised of type I IFN-STAT1-granzyme B is an important mechanism for up-regulation of NK cell cytotoxic activity following flu infection. This finding may provide an explanation for why NK cells from the STAT1-deficient animals have defective killing activity [62], [77], [78], considering the critical role of granzyme B in NK cell cytotoxicity [53], [79]. Our data also indicate that STAT4 signaling is dispensable for granzyme B induction by NK cells. In contrast, STAT4 signaling mediates IFN-γ production by NK cells in response to flu infection, consistent with previous reports using other pathogens [41], [54], [73], [80], suggesting that STAT4 activation for IFN-γ production is another key pathway of NK cell activation during flu infection. Consistent with the data obtained using the i.v. infection model, we observed that type I IFNs play an important role in activation of NK cell effector functions in the respiratory infection setting, indicating that our findings on the biochemical activation mechanisms are likely to be applicable to NK cell activation in the lungs. However, future studies are warranted for further understanding of NK cell activation in the lungs. Nonetheless, despite controversial reports (e.g. antiviral or pathological roles) on the importance of NK cells during flu infection, our study provides insights into the mechanisms of NK cell activation during influenza infection.

## Supporting Information

Figure S1 **C57BL/6 NK cells express effector molecules in response to influenza infection in vitro.** Splenocytes from B6 WT mice were cultured in vitro for indicated times in the presence or absence of influenza. Brefeldin A or monensin was added for the final 4 hours of each incubation period to inhibit effector molecule secretion. Histograms show percentages (inset values) of IFN-γ^+^ (upper panels) or granzyme B^+^ (lower panels) NK cells (NK1.1**^+^**CD3**^−^**CD19**^−^**).
(TIF)

Figure S2 **Type I IFNs are required for NK cell activation in response to flu infection.** Following i.v. infection with flu, splenic NK cells (NK1.1**^+^**CD3**^−^**CD19**^−^**) from B6 WT, IFNAR**^−/−^**, IL-12R**^−/−^** and IL-18R**^−/−^** mice were analyzed at 9 h post-infection. Graphs show percentages of NK cells expressing IFN-γ (left) and granzyme B (right). Each dot represents an individual mouse. Data are merged and the number of mice (n) is indicated for each group. Statistical analyses were performed using the unpaired Student’s t-test. **, *P*<0.001; ***, *P*<0.0001.
(TIF)

FIgure S3 **Type I IFNs are required for pulmonary NK cell activation in response to influenza infection.** B6 WT and IFNAR^−/−^ mice were infected intranasally (i.n.) with influenza for two days. Harvested lung tissue was digested with collagenase to release NK cells, then cells were analyzed by flow cytometry. Dot plots show IFN-γ^+^ (left panels) and granzyme B^+^ (right panels) (NK1.1**^+^**CD3**^−^**CD19**^−^**) NK cells. Values represent percentages of cells in the indicated quadrants. Data are representative of three independent experiments with 2–5 mice per group.
(TIF)

Figure S4 **Direct action of type I IFNs is critical for activation of NK cells following flu infection.** Splenocytes from IFNAR**^+/−^** or IFNAR**^−/−^** (CD45.2**^+^**) were transferred into CD45.1**^+^** B6 WT recipients by i.v. injection prior to infection with flu. Percentages of NK cells expressing IFN-γ (left) and granzyme B (right) were analyzed after transfer and infection. Each dot represents an individual mouse. Data are merged from four separate experiments and the number of mice (n) is indicated in each group. Statistical analyses were performed using the unpaired Student’s t-test. **, *P*<0.001; ***, *P*<0.0001.
(TIF)

Figure S5 **Phopho-STAT stainings are specific.** Splenocytes from B6 WT, STAT1^−/−^, STAT4^−/−^ and IFNAR^−/−^ were co-cultured with IFN-α (1,000 U/mL) for 30 m, then phospho-STAT level of NK cells was determined. Values represent the percentages of phospho-STAT**^+^** NK cells. Data are representative from at least 2 experiments.
(TIF)
