# Supplementary figures and images for: Activation Mechanisms of Natural Killer Cells during Influenza Virus Infection
Source: PLoS One. 2012 Dec 31;7(12):e51858. doi: 10.1371/journal.pone.0051858 (PMC3534084; doi:10.1371/journal.pone.0051858)

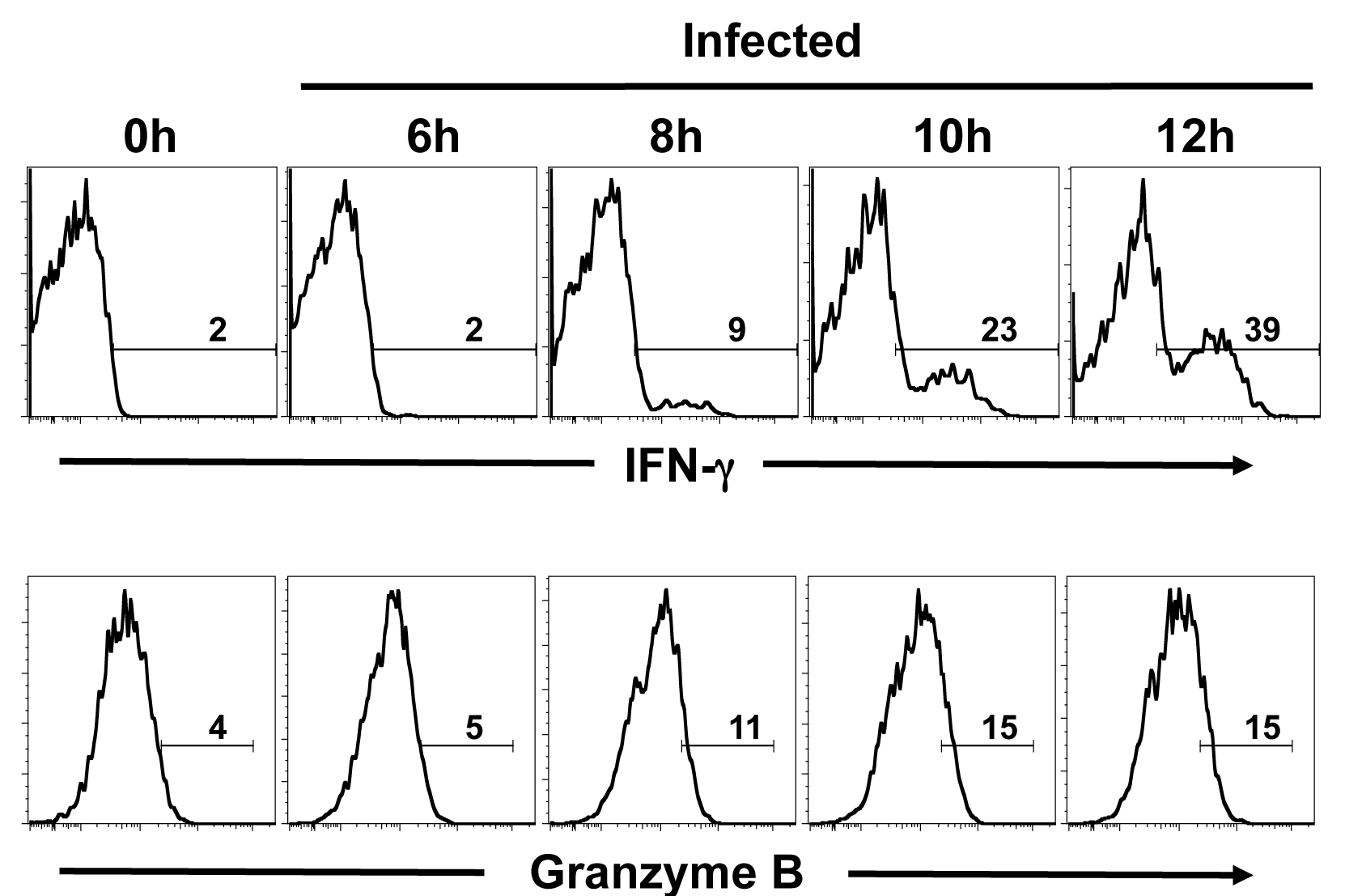

Supplement: Figure S1 — C57BL/6 NK cells express effector molecules in response to influenza infection in vitro. Splenocytes from B6 WT mice were cultured in vitro for indicated times in the presence or absence of influenza. Brefeldin A or monensin was added for the final 4 hours of each incubation period to inhibit effector molecule secretion. Histograms show percentages (inset values) of IFN-γ+ (upper panels) or granzyme B+ (lower panels) NK cells (NK1.1+CD3−CD19−). (TIF) [file pone.0051858.s001.tif]

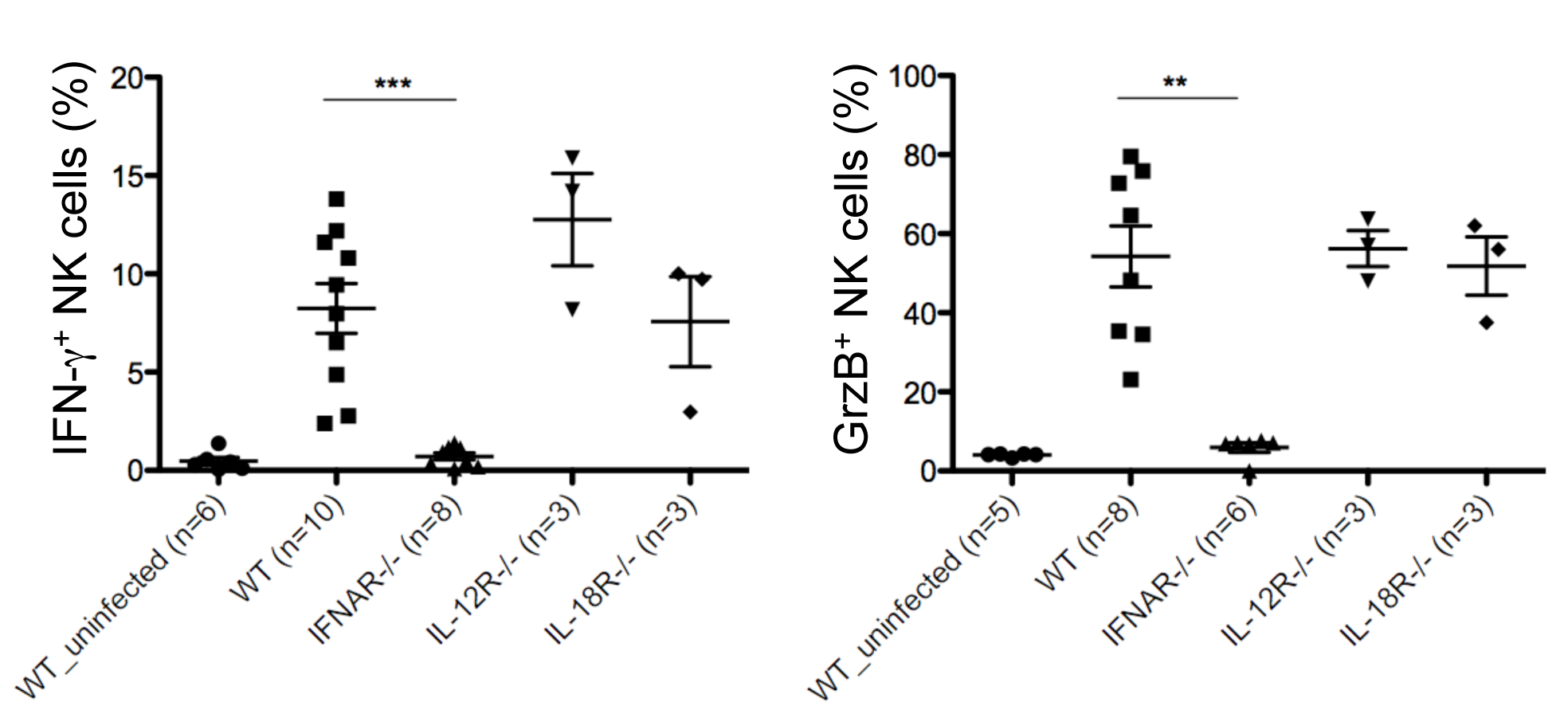

Supplement: Figure S2 — Type I IFNs are required for NK cell activation in response to flu infection. Following i.v. infection with flu, splenic NK cells (NK1.1+CD3−CD19−) from B6 WT, IFNAR−/−, IL-12R−/− and IL-18R−/− mice were analyzed at 9 h post-infection. Graphs show percentages of NK cells expressing IFN-γ (left) and granzyme B (right). Each dot represents an individual mouse. Data are merged and the number of mice (n) is indicated for each group. Statistical analyses were performed using the unpaired Student’s t-test. **, P<0.001; ***, P<0.0001. (TIF) [file pone.0051858.s002.tif]

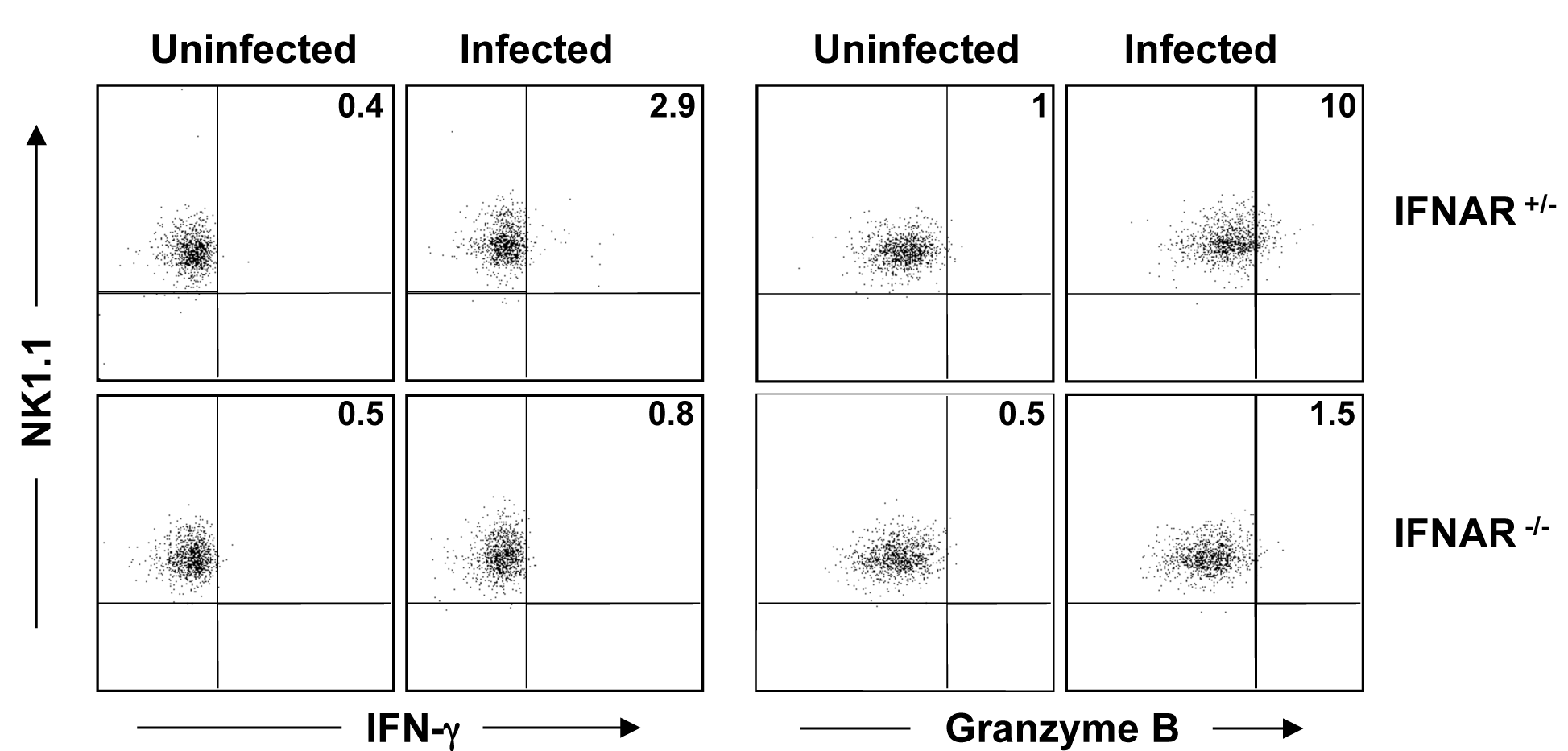

Supplement: FIgure S3 — Type I IFNs are required for pulmonary NK cell activation in response to influenza infection. B6 WT and IFNAR−/− mice were infected intranasally (i.n.) with influenza for two days. Harvested lung tissue was digested with collagenase to release NK cells, then cells were analyzed by flow cytometry. Dot plots show IFN-γ+ (left panels) and granzyme B+ (right panels) (NK1.1+CD3−CD19−) NK cells. Values represent percentages of cells in the indicated quadrants. Data are representative of three independent experiments with 2–5 mice per group. (TIF) [file pone.0051858.s003.tif]

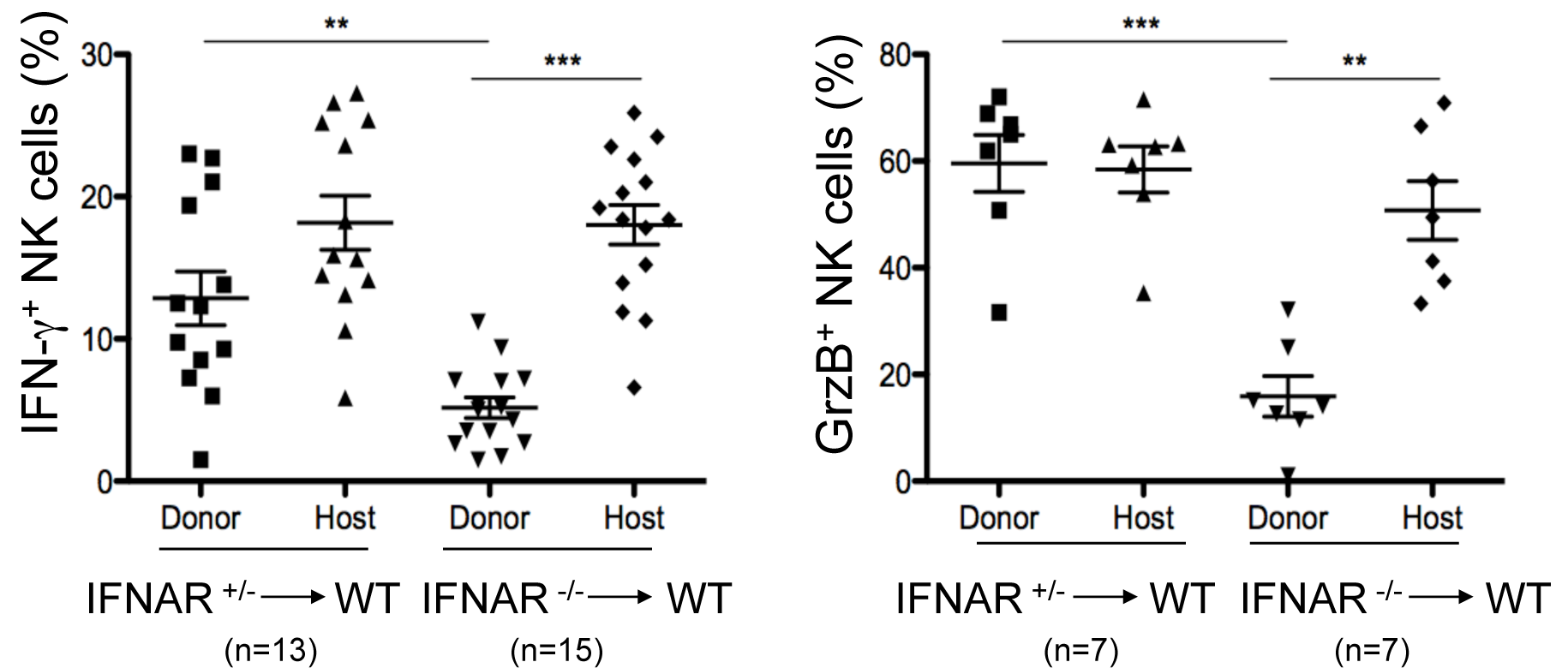

Supplement: Figure S4 — Direct action of type I IFNs is critical for activation of NK cells following flu infection. Splenocytes from IFNAR+/− or IFNAR−/− (CD45.2+) were transferred into CD45.1+ B6 WT recipients by i.v. injection prior to infection with flu. Percentages of NK cells expressing IFN-γ (left) and granzyme B (right) were analyzed after transfer and infection. Each dot represents an individual mouse. Data are merged from four separate experiments and the number of mice (n) is indicated in each group. Statistical analyses were performed using the unpaired Student’s t-test. **, P<0.001; ***, P<0.0001. (TIF) [file pone.0051858.s004.tif]

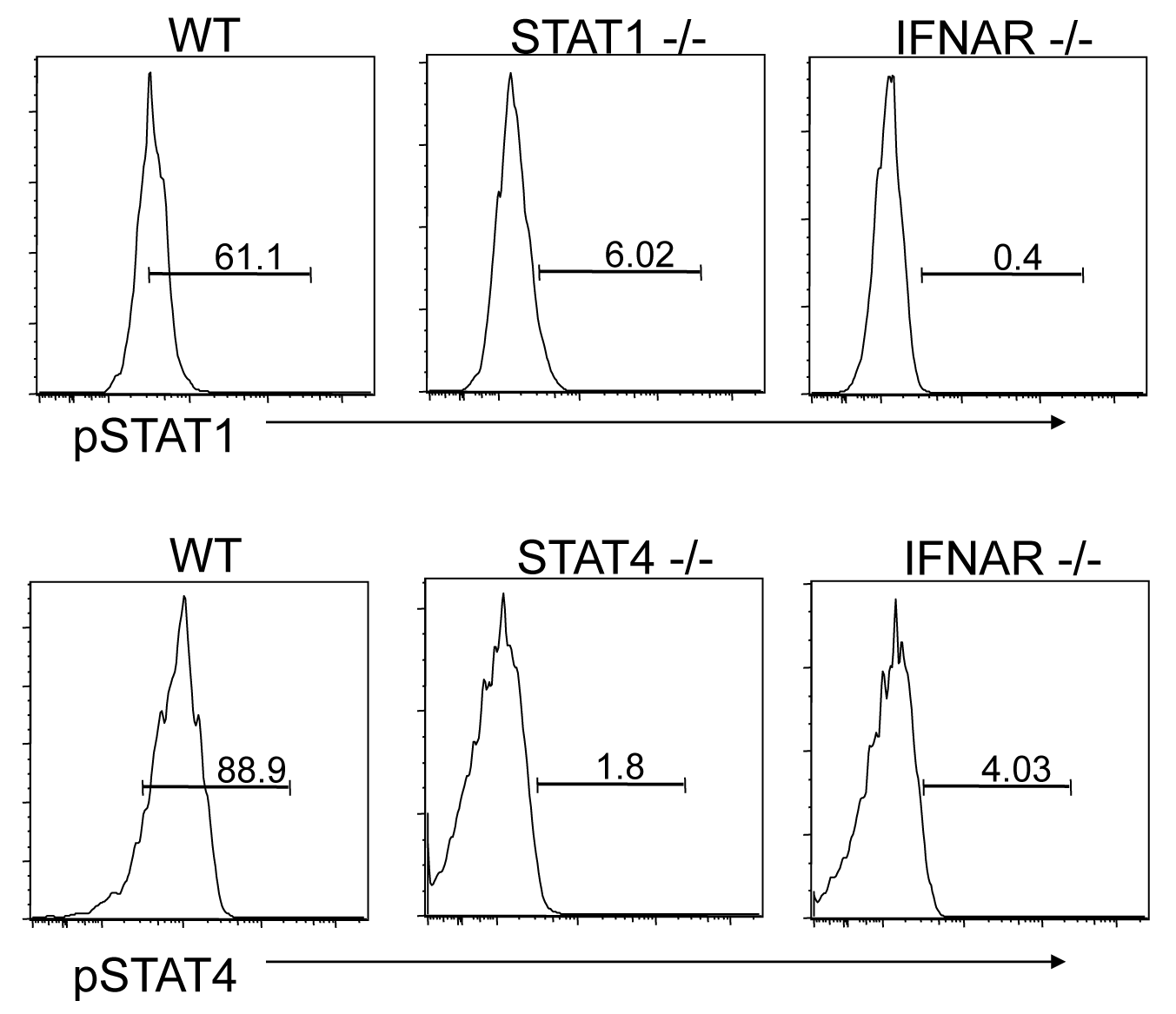

Supplement: Figure S5 — Phopho-STAT stainings are specific. Splenocytes from B6 WT, STAT1−/−, STAT4−/− and IFNAR−/− were co-cultured with IFN-α (1,000 U/mL) for 30 m, then phospho-STAT level of NK cells was determined. Values represent the percentages of phospho-STAT+ NK cells. Data are representative from at least 2 experiments. (TIF) [file pone.0051858.s005.tif]
